# Unravelling a couple in conflict: Undiagnosed obstructive sleep apnea

**DOI:** 10.1192/j.eurpsy.2021.2161

**Published:** 2021-08-13

**Authors:** I. Ganhao

**Affiliations:** Clinic 6, Centro Hospitalar Psiquiatrico de Lisboa, Lisbon, Portugal

**Keywords:** intimate partners, couple in conflict, obsrtuctive sleep apnea

## Abstract

**Introduction:**

Obstructive sleep apnea impacts quality of sleep and leads to sleep deprivation with consequences on level of general functioning and interpersonal relationships besides the known contribution to cardio and cerebrovascular disorders and sexual dysfunction. Most adults sleep with a partner and sleep disorders may also disrupt the partner´s sleep, yet surprisingly obstructive sleep apnea, even when such is the case, goes frequently undiagnosed.

**Objectives:**

To reflect on a clinical case that presents apparently as just another couple in conflict but in fact when unravelled leads to a diagnosis of obstructive sleep apnea that may have significant contribution to the conflict.

**Methods:**

Unravelling what was at the core of a couple in conflict.

**Results:**

A heterosexual couple in their late thities present in serious conflict with each other. Both are depressed and anxious, sleeping badly, with intimacy issues and having trouble dealing with work obligations and household chores. After continued squabbling, some self-reflection but mostly other blaming, a thread released by the female partner leads to sleep evaluation of the male partner with a resulting diagnosis of obstructive sleep apnea and subsequent treatment. And the couple relationship got better…

**Conclusions:**

Obstructive sleep apnea is frequent in the general population and more often than not undiagnosed but may be even more frequent in patients seeking mental health services. A sleep history is an important part of evaluation of patients who present with anxiety, depressive, sexual function and/or cognitive complaints and relationship issues. Interviewing intimate partners may provide essential clues to the possibility of existing sleep disorders.

**Disclosure:**

No significant relationships.

